# Low-latency stage-adaptive cascade architecture for real time non-stationary noise filtering

**DOI:** 10.1371/journal.pone.0354022

**Published:** 2026-07-22

**Authors:** Thanh Han-Trong, Thang Bui Van, Quang Hoang Minh, Anh Do Trung

**Affiliations:** 1 School of Electrical and Electronic Engineering, Hanoi University of Science and Technology, Hanoi, Vietnam; 2 Department of Science and Technology Management and International Cooperation, Posts and Telecommunications Institute of Technology, Hanoi, Vietnam; National Changhua University of Education, TAIWAN

## Abstract

In many real-time measurement and monitoring systems, the quality of acquired signals is often severely degraded by complex environmental noise sources with non-stationary properties, rendering analysis, important feature extraction, and decision-making unreliable. This study proposes a multi-stage adaptive denoising architecture based on the least mean square (LMS) algorithm, in which the number of filter stages and the step size are automatically adjusted according to error statistics, the remaining correlation between the residual and the reference signal, and the real-time signal-to-noise ratio (SNR) of the signal. The stopping mechanism is determined by a two-tailed Fisher-z correlation test, with effective sample size correction in the presence of autocorrelation and modulation based on SNR, to ensure the stability of the adaptive system against non-stationary noise. The filter is evaluated on simulated signal datasets and real-world measured data. Compared with the conventional LMS filter configuration under the tested simulated conditions, the proposed architecture reduces mean squared error (MSE) by 38–82% and mean absolute error (MAE) by 15–45%, while improving both SNR and peak signal-to-noise ratio (PSNR). The execution time of the proposed method is approximately 3.5–4 times lower than that of the fixed-threshold method under the tested settings. These results indicate that the proposed method can improve the trade-off between denoising performance and computational efficiency, showing potential for low-latency implementation on resource-constrained devices.

## 1. Introduction

In the context of the Internet of Things (IoT) and Cyber-Physical Systems (CPS), billions of sensors continuously generate real-time time-series signals to support monitoring in industrial systems, healthcare, transportation infrastructure, and smart cities [1–3]. In these systems, the quality of the acquired signals plays a crucial role in anomaly detection, diagnosis, decision-making and feedback control. However, in real-world environments, signals are often severely degraded by multiple non-stationary noise sources that vary rapidly in both amplitude and spectral characteristics. This leads to significant distortions in feature extraction, parameter estimation and decision-making processes at higher processing levels.

In parallel, the trend toward Edge AI (edge computing), rather than relying entirely on cloud infrastructure, is rapidly gaining momentum in order to reduce latency, save bandwidth and enhance data privacy in distributed CPS systems [2,4]– 6]. Many recent surveys show that Edge AI on IoT/IoMT platforms require signal processing algorithms and machine learning models to be very lightweight and low-power, while still maintaining reliable real-time performance [4–6]. This creates an urgent need for denoising techniques that are both effective and computationally efficient, making them suitable for microcontrollers and low-cost embedded devices.

Deep learning models have achieved considerable progress in signal denoising and signal quality enhancement. Many architectures such as CNNs, RNNs, LSTMs, U-Net, and Transformers have been successfully applied to denoising tasks in biomedical signals, speech, and vibration signals [7–11]. However, these models typically involve many parameters and require substantial memory and inference cost, making direct deployment on resource-constrained IoT/Edge sensors challenging, even when applying TinyML, model compression or quantization techniques [4– [6,10,11].

From another perspective, time-frequency signal decomposition methods such as the Wavelet Transform (WT), Empirical Mode Decomposition (EMD), Complete Ensemble EMD (CEEMD) and Variational Mode Decomposition (VMD), are still widely used for denoising nonlinear signals [12–15]. Although demonstrating effectiveness across a wide range of real-world signals, these methods typically require extensive manual tuning of hyperparameters, making them less flexible in the presence of rapidly varying noise and difficult to meet real-time processing requirements on resource-constrained hardware [12–15].

In scenarios that require continuous processing with low latency, adaptive filters, particularly the LMS family of algorithms, remain a suitable choice due to their simple linear structure, low computational cost, and capability for sample-by-sample updates [16–19]. However, conventional LMS architecture typically employs fixed parameters and stopping criteria, making it difficult to achieve a proper trade-off between convergence speed and stability when dealing with non-stationary noise or large-amplitude impulsive disturbances.

A significant limitation of these methods is the lack of a mechanism to control the filter structure based on clear statistical criteria, which makes them difficult to apply to non-stationary noise types. Recent results on time-series correlation have emphasized the role of the Fisher-z transformation in constructing confidence intervals for correlation coefficients, as well as the importance of adjusting the effective sample size (ESS) when autocorrelation is present in [20–22]. This suggests that using correlation tests on the residuals and reference signals could provide a solid basis for deciding when an adaptive filter should stop or switch configurations, rather than rely on heuristic thresholds.

On this basis, the present study proposes a method that uses a multi-stage adaptive denoising architecture based on the LMS algorithm, employing statistical foundations to determine decision thresholds that are robust to non-stationary noise in practical deployments. Adaptive noise cancellers (ANC) are applied for signal denoising due to their ability to adapt over time and their effectiveness against various types of noise [23–25], while also having low complexity, making them suitable for real-time and low-cost signal denoising applications [26]. The choice of a suitable algorithm for the architecture needs to provide low latency with minimal computational load and low cost. For algorithms such as NLMS [27], RLS [28], or Filtered-x LMS [29], although they perform well, they require high computational effort and cost. The use of the LMS algorithm meets the requirements for practical deployment on resource-constrained devices [30]. The use of a multi-stage architecture has also been shown to be more effective than a single stage [31] but using a fixed stopping threshold can easily lead to instability, over-filtering, or under-filtering under non-stationary noise conditions.

In this study, the stopping threshold is determined automatically based on clear statistical criteria so that the adaptive filtering architecture can adjust the number of filtering stages according to the remaining residual-reference correlation and local signal quality. The main contribution of this study is not the LMS filter itself, but the integration of a stage-adaptive LMS cascade with a Fisher-z-based stopping criterion, effective sample size correction for autocorrelated data, SNR-adaptive threshold modulation, and a final refinement stage for low-latency denoising. This design aims to improve the trade-off between denoising performance and computational efficiency, making it promising for implementation on resource-constrained devices such as low-cost MCUs or SoCs.

The remainder of the paper is organized as follows: the Related work sectionpresents an overview of related denoising approaches; the Methodology sectiondescribes in detail the proposed architectural framework and the theoretical basis of the Fisher-z mechanism and ESS; the Results sectionpresents and analyzes the experimental results; and the Conclusion section summarizes the study and discusses future directions.

## 2. Related work

With the rapid advancement and widespread global adoption of artificial intelligence technologies, the application of deep learning methods to signal denoising has become increasingly prevalent, showing strong potential in both research and practical applications across different domains. Many network architectures such as CNNs, autoencoders, U-Net, BiLSTM, and Transformers have been proposed for denoising tasks involving seismic, vibration, acoustic, and biomedical signals [32–35]. Experimental results show that these methods are capable of significantly improving signal quality and the SNR. Overall, approaches that use deep learning models for signal denoising show significant potential but require diverse training datasets to generalize well. These models have large numbers of parameters, high computational and memory costs, making it difficult to ensure real-time performance and low cost on resource-constrained devices [36,37].

To address the requirement for diverse training datasets in deep learning models, many studies have explored time-frequency domain representations or adaptive signal decomposition methods, followed by processing of the resulting coefficients. Wavelet-based methods, CEEMDAN, SVD, and Singular Spectrum Analysis (SSA) have been effectively applied to denoising vibration signals, heart sound signals, and acceleration signals [38–40]. In summary, the methods discussed all yield good results but incur high computational costs, with performance heavily dependent on hyperparameters and the selection of parameters such as the number of decomposition levels, rules, and threshold values, which are often chosen based on experience or trial for each type of noise, making them unstable when the noise changes. The lack of a statistically based stopping criterion and a time-adaptive mechanism can lead to performance degradation in the presence of non-stationary noise and changing environments.

In addition, adaptive filters are still regarded as suitable solutions for real-time denoising applications due to their simple structure and low computational cost [30,31]. However, most existing studies still rely on fixed stopping criteria and parameters, largely determined empirically, which leads to over-filtering or under-filtering when confronted with non-stationary and rapidly varying noise [30,31].

Therefore, this study introduces a statistically grounded stopping criterion and a time-adaptive mechanism for practical denoising applications.

## 3. Methodology

For clarity and consistency, the main notation used throughout the proposed adaptive denoising architecture is summarized below. In the revised manuscript, distinct symbols are used for the clean signal, noise component, reference noise signal, residual output, and correlation coefficient to avoid ambiguity in the mathematical formulation and algorithm description.

### Notation Summary

**Table:** 

Symbol	Definition	Symbol	Definition
P[n]	Measured/composite signal	$\mu_{i}$	Step size at stage i
c[n]	Clean signal	$N_{w}$	Window length
r[n]	Noise component	m	Window index
$r^{\prime }[n]$	Reference noise signal	${\;\hat{\rho }}_{i,m\;}$	Residual-reference correlation
${{𝐫}^{\prime }}_{\text{n}}$	Reference-noise input vector	z	Fisher-z transformed correlation
M	LMS filter order	$N_{\text{eff}}$	Effective sample size
i	Stage index in Module I	$\alpha_{\text{dyn}}$	Dynamic significance level
j	Selected final stage in Module I	$\rho_{\text{th}}$	Dynamic correlation threshold
$x_{i}[n]$	Reference-noise-related input at stagei	${\text{SNR}}_{i,m}$	Window-based SNR
$P_{i}[n]$	Input signal of stagei	K	Consecutive-window count
$y_{i}[n]$	Estimated noise at stagei	$J_{max}$	Maximum number of stages in Module I
$e_{i}[n]$	Residual/error output at stagei	$\tilde{c}[n]$	Preliminary clean-signal estimate from Module I
${𝐰}_{i}[n]$	LMS coefficient vector at stagei	$\hat{c}[n]$	Final denoised output after final filtering stage

For clarity, P[n] denotes the measured composite signal, which consists of the desired clean signal c[n] and the noise component r[n]. The input of the first adaptive filtering stage is $P_{\text{1}}[n]=P[n]$, and the residual output of each stage becomes the input of the next stage, $P_{i+\text{1}}[n]=e_{i}[n]$. Here, i denotes the current adaptive filtering stage in Module I, whereas j denotes the final stage automatically selected by the proposed stopping rule. Thus, i=1,2,…,j, with $j\leq J_{\text{max}}$. The final filtering stage is not included in j. The notation ${\hat{\rho }}_{i,m\;}$is used for the Pearson residual-reference correlation coefficient in window m at stage i, in order to avoid confusion with the noise component r[n] and the reference noise signal $r^{\prime }[n]$.

After Module I stops, the residual output at the selected stage is denoted by $\tilde{c}[n]$, where $\tilde{c}[n]=e_{j}[n].$ Here, $\tilde{c}[n]\;$represents a preliminary clean-signal estimate, not the true clean signal. The final denoised output after the final filtering stage is denoted by $\hat{c}[n]$.

### 3.1 Architecture

In this study, we propose a multi-stage adaptive denoising architecture based on the LMS algorithm, with statistically determined thresholds modulated according to the SNR. The method follows the adaptive noise cancellation framework and uses two input channels: the measured composite signal P[n] and the reference noise signal $r^{\prime }[n]$. The measured signal is modeled as P[n]=c[n]+r[n], where c[n] is the desired clean signal and r[n] is the noise component. The reference noise signal $r^{\prime }[n]\;$is assumed to be correlated with r[n]and weakly correlated or uncorrelated with c[n].

At stage i in Module I, the LMS filter estimates the noise component in $P_{i}[n]$ using the reference-noise input vector ${𝐫}_{n}^{\prime }$. The residual output is computed as $e_{i}[n]=P_{i}[n]-y_{i}[n],$ where $y_{i}[n]\;$is the estimated noise at stage i. If another stage is required, the residual becomes the next-stage input:$P_{i+\text{1}}[n]=e_{i}[n].$

The step size $\mu_{i}$is adjusted at each stage to balance convergence speed, low latency, and stability.

**Fig 1:**
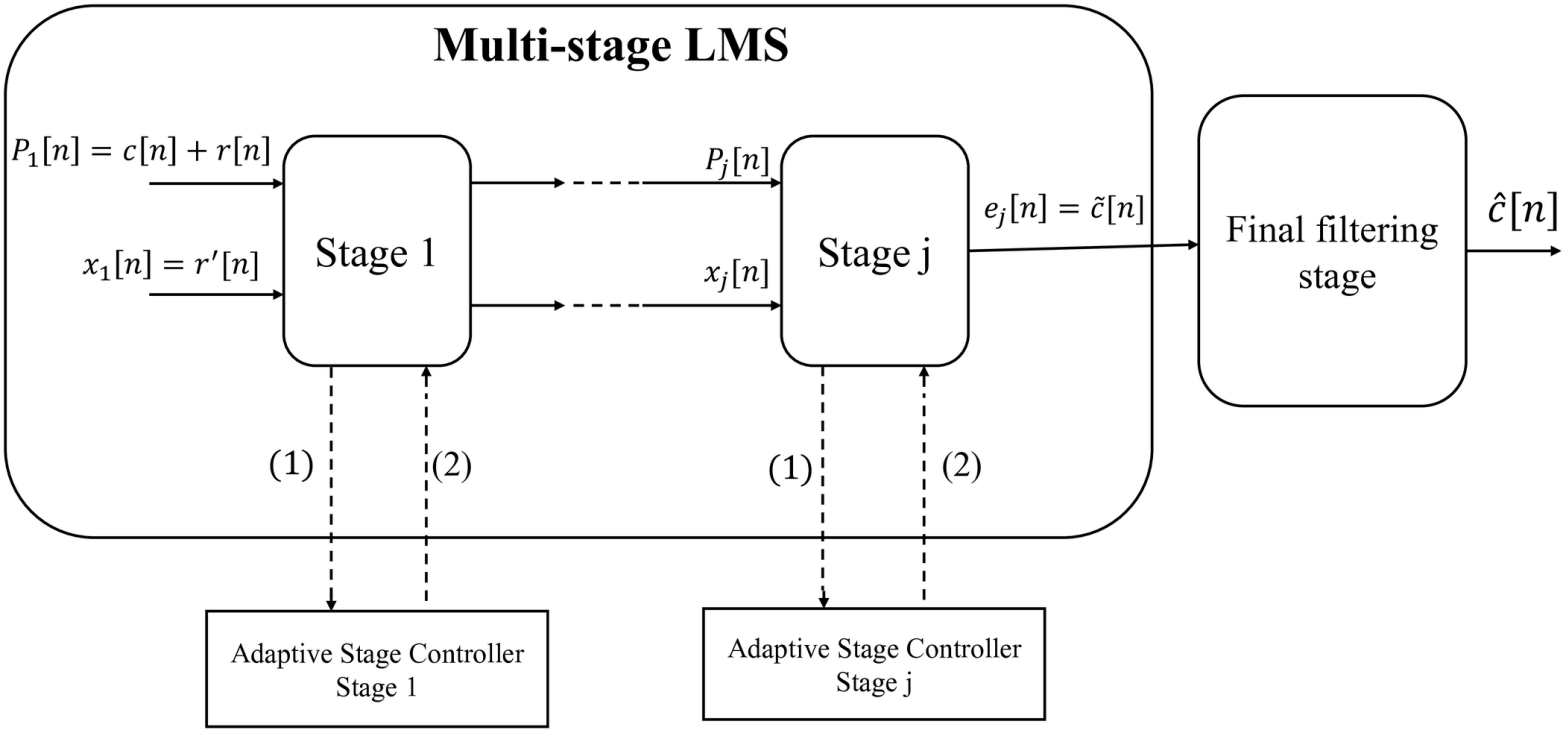
Proposed adaptive denoising architecture.

**Fig 2:**
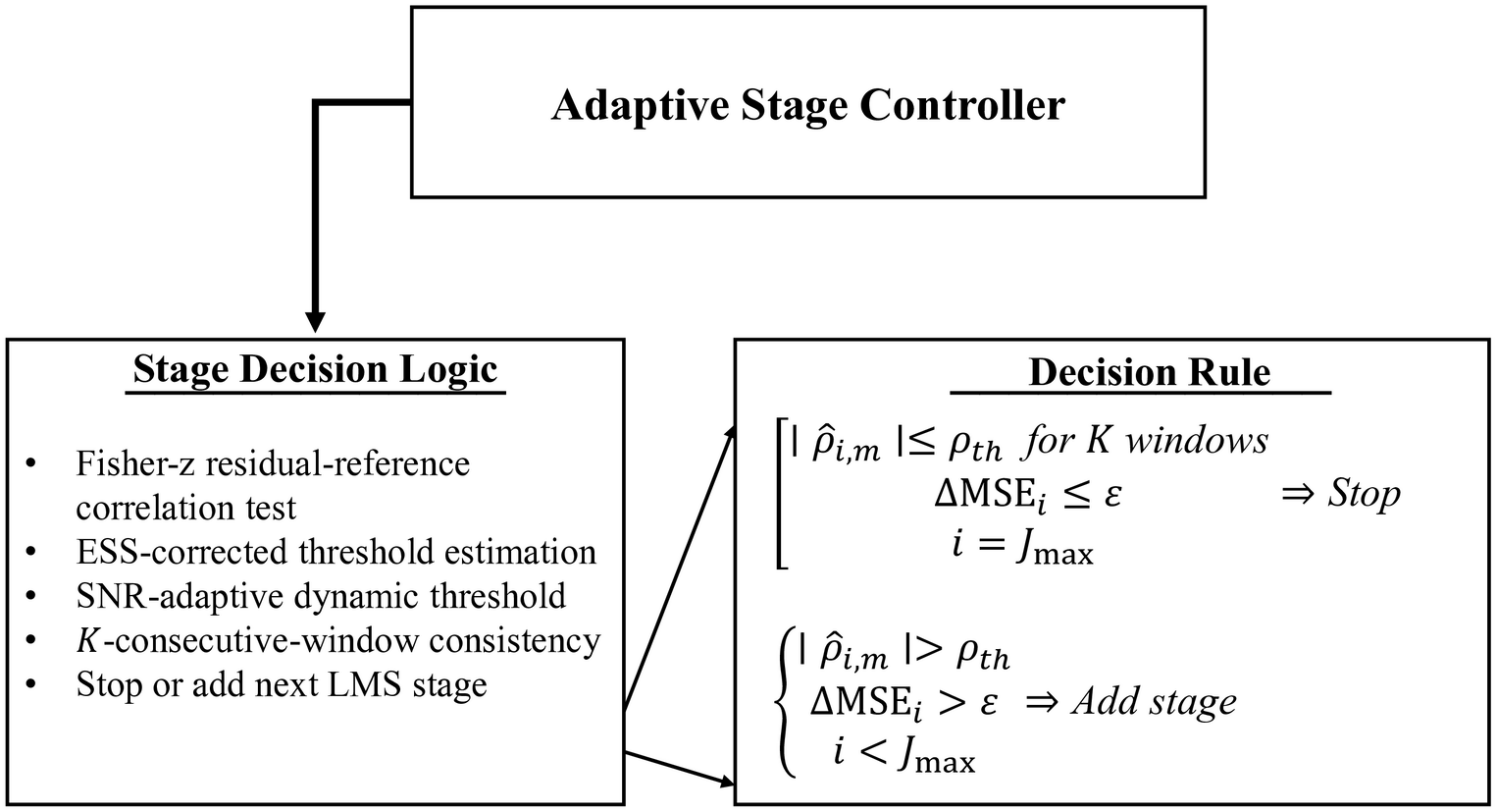
Adaptive stage controller and stage decision logic.

The proposed denoising architecture is illustrated in Fig 1. In this framework, the input signal is processed sequentially through cascaded LMS stages. Each stage utilizes the reference noise signal to estimate and suppress the correlated noise component within the current stage input, while simultaneously generating a residual signal that serves as the input for the subsequent stage. Unlike conventional multi-stage LMS architectures that employ a fixed number of stages or fixed stopping thresholds, this study proposes an adaptive stage control mechanism that automatically determines whether to stop or to add an additional filtering stage. This control mechanism is illustrated in Fig 2 as the Adaptive Stage Controller. At each stage, the controller uses only quantities that are available during online operation, including the residual-reference correlation, the estimated window-based SNR, and the marginal change in the residual-reference indicator. Reference-based metrics such as MSE, MAE, SNR, and PSNR are used only for offline evaluation on simulated data where the clean reference signal is available, and are not used by the stage-control mechanism.

(1) Stats (${\hat{\rho }}_{i,m\;},\;{\text{SNR}}_{i,m},\;{\Delta \text{MSE}}_{i}$)

(2) Stage control decision

The number of denoising stages is automatically determined by a statistical stopping criterion, specifically a two-sided Fisher-z correlation test between $e_{i}$ and the reference signal over sliding time windows, with effective sample size correction in the presence of autocorrelation and modulation according to the window-based SNR [41–43]. The stage addition process terminates when the correlation is no longer statistically significant, indicating that the post-filtering error no longer contains noise components that can be estimated from the reference channel; therefore, adding further filtering stages would not provide additional noise reduction and may risk distorting the clean signal.

The detailed signal relationships at the selected final stage j of Module I are described below.

For the ANC-based denoising system, two input channels are required: the primary signal channel to be filtered and a reference noise channel that is correlated with the noise present in the primary signal. For the first stage, the inputs consist of the composite signal $P_{\text{1}}[n]$, which is the measured signal comprising the clean signal c[n] and noise r[n], and the reference noise signal $r^{\prime }[n]$, which is correlated with the noise component r[n] in the composite signal.

In the following derivation, we focus on the selected final stage j of Module I. Thus, $P_{j}[n]$, $x_{j}[n]$, $y_{j}[n]$, and $e_{j}[n]\;$denote the stage-wise quantities at the final stage selected by the stopping rule. The inputs at stage j are given by


$$\begin{array}{c} P_{j}[n]=\;e_{j-\text{1}}[n]=c[n]+\;\rho_{j}r[n] \end{array}$$ (1)

$$\begin{array}{c} x_{j}[n]=\;x_{j-\text{1}}[n]-\;y_{j-\text{1}}[n]\approx \rho_{j}r^{\prime }[n] \end{array}$$ (2)

Accordingly, the estimated interference at stage j is:


$$\begin{array}{c} y_{j}[n]=\;{𝐰}_{j}^{T}[n]x_{j}[n]\approx \;{𝐰}_{j}^{T}\rho_{j}r^{\prime }[n]\;\approx \;\rho_{j}\hat{r}[n] \end{array}$$ (3)

The weight vector is updated as:


$$\begin{array}{c} {𝐰}_{j}[n+\text{1}]=\;{𝐰}_{j}[n]+\;\mu_{j}e_{j}[n]x_{j}[n] \end{array}$$ (4)

Here, $\rho_{j}\in [\text{0},\text{1}]\;$denotes the amplitude ratio of the remaining correlated noise component at the selected final stage j. This parameter is not the Pearson residual-reference correlation coefficient ${\hat{\rho }}_{i,m\;}$used in the stopping test.

The residual/error output at stage j is:


$$\begin{array}{c} e_{j}[n]=P_{j}[n]-\;y_{j}[n]\approx c[n]+\;\rho_{j}r[n]-\;\rho_{j}\hat{r}[n \end{array}$$ (5)

When the LMS filter converges, $\rho_{j}\hat{r}[n]$approximates the remaining correlated noise component $\rho_{j}r[n]$, and therefore $e_{j}[n]\approx c[n]$. Thus, the residual output at the selected final stage is treated as the preliminary clean-signal estimate of Module I: $\tilde{c}[n]=e_{j}[n].\;$This estimate is not the true clean signal; it is the intermediate clean-signal estimate used by the final filtering stage.

To refine the denoising result, the preliminary clean-signal estimate obtained from Module I is used as the reference input of the final filtering stage.

Accordingly, the two inputs of the final filtering stage are defined as:


$$\begin{array}{c} d_{F}[n]=P[n] \end{array}$$ (6)

$$\begin{array}{c} q_{F}[n]=\tilde{c}[n]=e_{j}[n] \end{array}$$ (7)

where $d_{F}[n]\;$is the primary input of the final filtering stage and $q_{F}[n]$ is the reference input constructed from the preliminary clean-signal estimate.

Let ${𝐪}_{F,n}$ denote the reference-input vector constructed from $q_{F}[n]$. The output of the final filtering stage is defined as:


$$\begin{array}{c} \hat{c}[n]={𝐰}_{F}^{T}[n]{𝐪}_{F,n} \end{array}$$ (8)

where $\hat{c}[n]\;$is the final denoised output. The corresponding error signal is given by


$$\begin{array}{c} e_{F}[n]=d_{F}[n]-\hat{c}[n] \end{array}$$ (9)

Thus, the final filtering stage is used only as a refinement stage after Module I has selected the final stage j. It is not included in the automatic stage-addition process, and it does not use the true clean signal as the reference input.

### 3.2 Step size

The step size μ directly determines the convergence speed and stability of the system. A large step size enables rapid adaptation but also results in a large excess mean square error (EMSE), which may lead to system instability. A small step size improves system stability but leads to slow convergence. In [44], Bismor *et al.* reviewed variable step-size LMS algorithms. The selection of the lower bound $\mu_{\text{min}}$ can be made simply and easily, as it does not significantly affect the system performance. However, the selection of the upper bound $\mu_{\text{max}}\;$requires careful consideration to avoid system instability. The upper bound should adapt to the instantaneous input energy, as expressed by the following equation:


$$\begin{array}{c} \mu_{max}(n)=\;\varepsilon \frac{\text{2}}{x^{T}(n)x(n)} \end{array}$$ (10)

where 0<ε<1 is a safety scaling factor used to keep the LMS step size below the theoretical stability bound. In this study, ε is selected in the range of 0.8–0.9 to provide a practical compromise between convergence speed and stability. A smaller value of ε increases the stability margin but may slow down convergence, whereas a larger value accelerates adaptation but may increase the risk of excess mean-square error or instability. This normalized step-size setting also reduces the number of hyperparameters to be tuned and maintains robustness under varying signal amplitudes, making it suitable for real-time applications.

Based on this principle, Pauline *et al.* applied it to a multi-stage adaptive filtering architecture [31], in which the step size is updated independently for each stage, ensuring more stable step-size behavior compared to using a fixed step size across all stages:


$$\begin{array}{c} \text{0}<\;\mu <\mu_{max}=\frac{\text{2}}{\lambda_{max}} \end{array}$$ (11)

where $\lambda_{\text{max}}$ denotes the largest eigenvalue of the input autocorrelation matrix.

With the upper bound of the step size chosen as in (11), the step size $\mu_{i}\;$at stage i is determined as follows:


$$\begin{array}{c} \mu_{i}=\;\left\{ \begin{array}{c} @l\mu_{i-\text{1}}*f,\;\mu_{{max}_{i}}>\mu_{{max}_{i-\text{1}}}\;\\ \frac{\mu_{i-\text{1}}}{f},\;{\;\mu }_{{max}_{i}}<\mu_{{max}_{i-\text{1}}}\\ \;\mu_{i-\text{1}},\;{\;\mu }_{{max}_{i}}=\mu_{{max}_{i-\text{1}}} \end{array}\right. \end{array}$$ (12)

where 1≤f≤2. The selection of the value of f is also important for LMS convergence but is relatively simple and not highly sensitive, helping to reduce the number of hyperparameters that need to be tuned.

This approach enables the LMS algorithm to converge rapidly while maintaining stage-wise system stability, ensuring effective real-time operation at low computational cost, and making it well suited for the resource-constrained devices targeted in this study.

### 3.3 Proposed automatic stage addition mechanism

The automatic stage addition mechanism determines whether the cascade should stop at the current stage or add another LMS stage. At each stage i in Module I, the residual/error output $e_{i}[n]$ is compared with the reference noise signal $r^{\prime }[n]$ over sliding windows. For window m with length $N_{w}$, the Pearson residual-reference correlation coefficient is denoted by ${\hat{\rho }}_{i,m}$.

A large value of $|{\hat{\rho }}_{i,m}|$ indicates that the residual output still contains a noise component correlated with the reference channel; therefore, an additional filtering stage may still be useful. Conversely, a statistically insignificant or very small value of $|{\hat{\rho }}_{i,m}|$ indicates that further filtering may provide little additional noise reduction and may increase the risk of over-filtering.

The signal is divided into windows of length $N_{w}$. Within window m, the Pearson residual-reference correlation coefficient is computed as


$$\begin{array}{c} {\hat{\rho }}_{i,m}=\frac{\sum_{n\in W_{m}}\left(e_{i}[n]-{\bar{e}}_{i,m}\right)\left(r^{\prime }[n]-{\bar{r}}_{m}^{\prime }\right)}{\sqrt{\sum_{n\in W_{m}}{\left(e_{i}[n]-{\bar{e}}_{i,m}\right)}^{\text{2}}}\sqrt{\sum_{n\in W_{m}}{\left(r^{\prime }[n]-{\bar{r}}_{m}^{\prime }\right)}^{\text{2}}}} \end{array}$$ (13)

where $W_{m}\;$denotes the set of samples in window m, ${\bar{e}}_{i,m}\;$is the mean value of $e_{i}[n]\;$in window m, and ${\bar{r}}_{m}^{\prime }\;$is the mean value of $r^{\prime }[n]\;$in the same window.

The hypothesis testing is formulated as follows:

$H_{\text{0}}:\;\rho_{i,m}=\text{0}$ (no statistically significant correlation remains)

$H_{\text{1}}:\;\rho_{i,m}\neq \text{0}$ with significance level $\alpha_{\text{0}}$, where $\rho_{i,m}\;$denotes the population correlation coefficient corresponding to the sample estimate ${\hat{\rho }}_{i,m}$.

The sample correlation coefficient ${\hat{\rho }}_{i,m}\;$ does not follow a normal distribution, its variance depends on the population correlation coefficient $\rho_{i,m}$, and it is bounded within [−1,1]. Therefore, a reliable threshold cannot be directly applied to ${\hat{\rho }}_{i,m}\;$. In this study, the Fisher-z transformation is employed to effectively approximate normality and stabilize the variance. The Fisher-z transformation converts ${\hat{\rho }}_{i,m}\;$ into:


$$\begin{array}{c} z_{i,m}=\frac{\text{1}}{\text{2}}\text{ln}\left(\frac{\text{1}+{\hat{\rho }}_{i,m}}{\text{1}-{\hat{\rho }}_{i,m}}\right)=\text{artanh}({\hat{\rho }}_{i,m}) \end{array}$$ (14)

Then, the Fisher-z variable is given by:


$$\begin{array}{c} z_{i,m}\sim N\left(\text{0},\frac{\text{1}}{N_{w}-\text{3}}\right) \end{array}$$ (15)

However, time -series data often exhibits autocorrelation, meaning that the effective number of independent observations is smaller than the window length $N_{w}$, which increases the likelihood of Type I errors and results in overly narrow confidence intervals.

Therefore, the effective sample size $N_{\text{eff}}\;$is used to correct the Fisher-z test and obtain a more reliable threshold [42]. The effective sample size is determined as follows:


$$\begin{array}{c} N_{\text{eff}}\approx \frac{N_{w}}{\text{1}+\text{2}\sum_{k=\text{1}}^{N_{w}-\text{1}}\left(\text{1}-\frac{k}{N_{w}}\right)\rho_{e}(k)\rho_{r^{\prime }}(k)} \end{array}$$ (16)

where $\rho_{e}(k)$ and $\rho_{r^{\prime }}(k)\;$denote the lag-k autocorrelation coefficients of $e_{i}[n]\;$and $r^{\prime }[n]$, respectively, within the analyzed window.

In practice, (16) is commonly approximated under an AR(1) model, using the following first-order autocorrelation coefficients [42]:


$$\begin{array}{c} N_{e\text{ff}}\;\approx N_{w}.\frac{\text{1}-\rho_{e}(\text{1})\rho_{r^{\prime }}(\text{1})}{\text{1}+\rho_{e}(\text{1})\rho_{r^{\prime }}(\text{1})} \end{array}$$ (17)

In addition, to mitigate the risk of over-filtering, we further adopt a dynamic significance level $\alpha_{\text{dyn}}$ that is adjusted according to the window-based SNR. This keeps the test more conservative under low-SNR conditions, while allowing faster adaptation when the residual signal quality is higher. The dynamic significance level is expressed as


$$\begin{array}{c} \alpha_{\text{dyn}}=\;\alpha_{\text{0}}e^{-\text{0}.\text{1}{SNR}_{i,m}} \end{array}$$ (18)

where the window-based SNR at stage i and window m is computed as


$$\begin{array}{c} {\text{SNR}}_{i,m}=\text{1}\text{0}{\text{log}}_{\text{1}\text{0}}\frac{E[e_{i}^{\text{2}}]}{E[y_{i}^{\text{2}}]} \end{array}$$ (19)

In this way, the significance level of the test is adjusted according to the local SNR condition, while the threshold is modulated to reduce Type I error and threshold fluctuations [45,46].

With the effective sample size $N_{\text{eff}}\;$and the dynamic significance level $\alpha_{\text{dyn}}$, the two-sided Fisher-z threshold is determined as follows:


$$\begin{array}{c} z^{\prime }=\;\frac{z_{\text{1}-\alpha_{\text{dyn}}/\text{2}}}{\sqrt{N_{\text{eff}}-\text{3}}} \end{array}$$ (20)

Accordingly, the dynamic correlation threshold is obtained by transforming the Fisher-z threshold back to the correlation domain:


$$\begin{array}{c} \rho_{th}=\text{tanh}\left(z^{\prime }\right)=\text{tanh}\left(\frac{z_{\text{1}-\alpha_{\text{dyn}/\text{2}}}}{\sqrt{N_{\text{eff}}-\text{3}}}\right) \end{array}$$ (21)

Thus, $\rho_{\text{th}}\;$is not a fixed empirical threshold, but is dynamically determined from the effective sample size and the local SNR condition.

In addition, we further incorporate a stopping condition based on ΔMSE, as defined by the following equation:


$$\begin{array}{c} {\Delta MSE}_{i}=\;\frac{{MSE}_{i-\text{1}}-{MSE}_{i}}{{MSE}_{i-\text{1}}}\;\leq \;\varepsilon \end{array}$$ (22)

where ε represents the marginal improvement threshold.

The stopping rule is defined by either of the following conditions: (i) when $|{\hat{\rho }}_{i,m}|\leq \rho_{\text{th}}\;$ for K consecutive windows, or (ii) when the marginal improvement is no longer significant (${\Delta MSE}_{i}\leq \varepsilon$). In this study, K=3 is used to reduce the risk of making a stopping decision based on a temporary correlation drop in a single window. A smaller K makes the system more responsive but less stable, whereas a larger K makes the decision more conservative but may increase latency.

When either stopping condition is satisfied at stage i, the selected final stage of Module I is set as j=i. If neither condition is satisfied, an additional LMS stage is added until the stopping condition is met or the maximum number of Module-I stages $J_{\text{max}}$ is reached. In this study, $J_{\text{max}}=\text{3}\;$is used as a practical upper bound for the automatic stage-addition process, following the three-stage cascade structure adopted in the multistage LMS framework. The additional final filtering stage is applied separately as a refinement stage and is not included in $J_{\text{max}}$. This prevents indefinite stage growth, limits computational cost, and reduces the risk of over-filtering. Therefore, the proposed mechanism can adapt to non-stationary noise while avoiding unnecessary additional stages.

## 4 Results

In this study, the filter performance is evaluated using two types of data, including simulated signal data and real-world measured data.

For simulated data, a clean reference signal is available; therefore, reference-based metrics, including MSE, MAE, SNR, PSNR, and CC, are used to evaluate reconstruction error, noise suppression, and waveform preservation. In contrast, for experimentally acquired PCG data, no clean reference signal is available, so MSE, SNR, and PSNR cannot be directly computed in the same manner. Therefore, the real-world evaluation relies on reference-free or noise-reference-based indicators, such as residual-reference correlation, magnitude-squared coherence, and signal quality indices, to assess noise reduction and PCG signal preservation.

The reference-based metrics for the simulated data are defined as follows. Here, c[n] denotes the clean reference signal, $\hat{c}[n]$ denotes the final denoised output after the final filtering stage, and N denotes the total number of samples used for performance evaluation.

Mean Squared Error (MSE)


$$\begin{array}{c} \text{MSE}=\frac{\text{1}}{N}\sum_{n=\text{1}}^{N}{\left(c[n]-\hat{c}[n]\right)}^{\text{2}} \end{array}$$ (23)

Signal-to-Noise Ratio (SNR) (dB)


$$\begin{array}{c} \text{SNR}=\text{1}\text{0}{\text{log}}_{\text{1}\text{0}}\left(\frac{\sum_{n=\text{1}}^{N}c^{\text{2}}[n]}{\sum_{n=\text{1}}^{N}{\left(c[n]-\hat{c}[n]\right)}^{\text{2}}}\right) \end{array}$$ (24)

Peak Signal-to-Noise Ratio (PSNR) (dB)


$$\begin{array}{c} \text{PSNR}=\text{1}\text{0}{\text{log}}_{\text{1}\text{0}}\left(\frac{\underset{\text{1}\leq n\leq N}{\text{max}}|c[n]{|}^{\text{2}}}{\text{MSE}}\right) \end{array}$$ (25)

Mean Absolute Error (MAE)


$$\begin{array}{c} \text{MAE}=\frac{\text{1}}{N}\sum_{n=\text{1}}^{N}|c[n]-\hat{c}[n]| \end{array}$$ (26)

Correlation Coefficient (CC)


$$\begin{array}{c} \text{CC}=\frac{N\sum_{n=\text{1}}^{N}c[n]\hat{c}[n]-\left(\sum_{n=\text{1}}^{N}c[n]\right)\left(\sum_{n=\text{1}}^{N}\hat{c}[n]\right)}{\sqrt{\left[N\sum_{n=\text{1}}^{N}c^{\text{2}}[n]-{\left(\sum_{n=\text{1}}^{N}c[n]\right)}^{\text{2}}\right]\left[N\sum_{n=\text{1}}^{N}{\hat{c}}^{\text{2}}[n]-{\left(\sum_{n=\text{1}}^{N}\hat{c}[n]\right)}^{\text{2}}\right]}} \end{array}$$ (27)

It should be noted that the correlation coefficient in Eq. (27) is well defined only when both the clean reference signal c[n] and the final denoised signal $\hat{c}[n]$ have non-zero variance over the evaluated samples. If either sequence is constant or has zero variance, the denominator of Eq. (27) becomes zero and the CC is undefined. In such cases, CC should not be reported, and reference-error metrics such as MSE or MAE should be used instead.

### 4.1 Simulated signal data

To evaluate the performance of the denoising filter under various operating conditions, we use a simulated signal dataset based on a clean sinusoidal signal and PCG signal. The added noise signals correspond to common types of noise encountered in measurement and signal processing systems, including Gaussian noise, low-frequency-dominant noise, Pink noise and combined noise.

**Fig 3:**
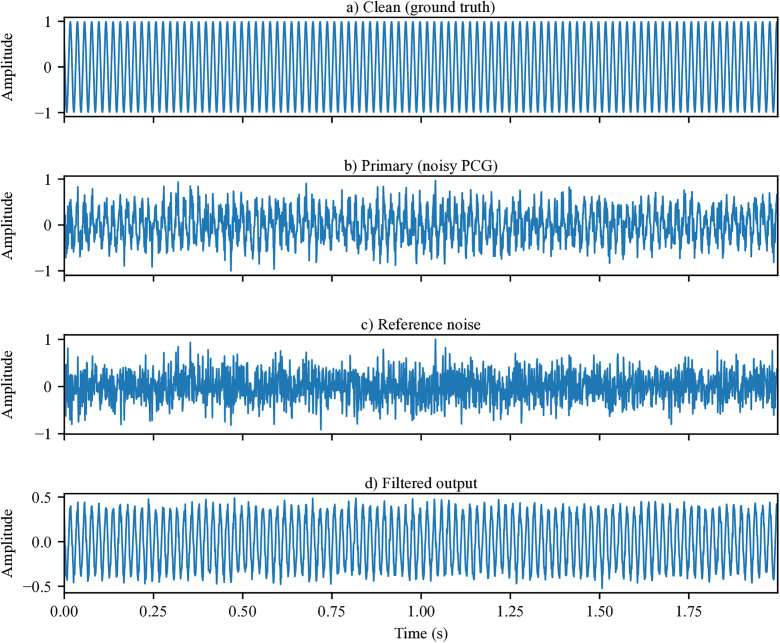
Performance of the proposed filter for sinusoidal signal with Gaussian noise. (a) Clean signal (b) Primary signal (c) Noise signal (d) Filtered signal.

In the case of Gaussian noise, as shown in Fig 3, the noise component exhibits an energy distribution that is spread across the entire frequency band, causing the composite received signal in Fig 3(b) to be masked by random fluctuations, which makes the sinusoidal waveform difficult to identify and analyze. The denoising result shown in Fig 3(d) demonstrates the ability to effectively reconstruct the clean signal, achieve strong noise suppression, closely follow the original signal waveform and exhibit a clear improvement in signal cleanliness compared to the input signal. This indicates the effectiveness of the proposed method under broadband random noise conditions. The improvement in Fig 3 is mainly reflected by the reduction of high-frequency random fluctuations around the sinusoidal signal. The filtered signal preserves the phase and amplitude trend of the clean signal, which is consistent with the high correlation coefficient reported in the quantitative results.

**Fig 4:**
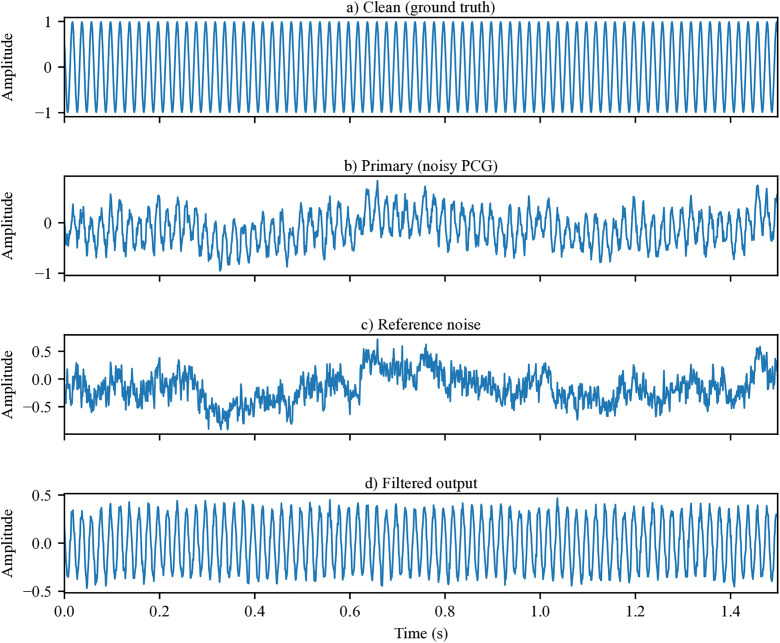
Performance of the proposed filter for composite signal with low-frequency dominant noise. (a) Clean signal (b) Primary signal (c) Noise signal (d) Filtered signal.

For the noise dominated by low frequency components shown in Fig 4, the clean sinusoidal signal is masked by slow fluctuations whose amplitude varies over time. This type of noise significantly alters the signal envelope and causes a pronounced baseline drift, as illustrated in Fig 4(b). When ${SNR}_{in}$ is low, the overlap between the noise and the clean signal further reduces the ability to identify the harmonic waveform. The result shown in Fig 4(d) indicates that the denoising filter is still able to effectively reconstruct the basic periodic signal structure, while the background noise energy is significantly reduced compared to the input signal, leading to a clear reduction in the baseline drift observed in Fig 4(b). This result suggests that the proposed filter can suppress slowly varying components that behave as baseline drift. The preservation of the main periodic waveform indicates that the adaptive cascade does not simply attenuate the whole low-frequency content, but reduces the reference-correlated disturbance while retaining the useful signal structure.

**Fig 5:**
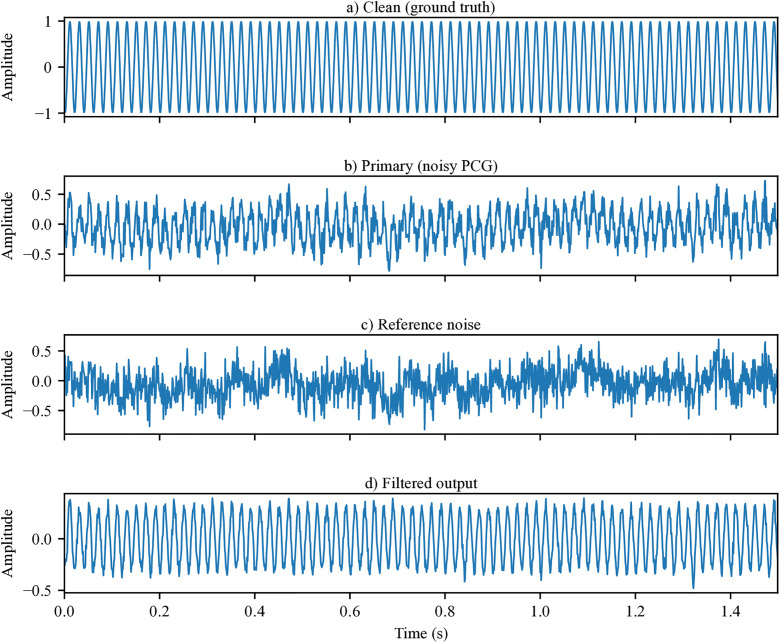
Performance of the proposed filter for composite signal with combined noise (a) Clean signal (b) Primary signal (c) Noise signal (d) Filtered signal.

In practice, the noise source is a combination of multiple noise types, as shown in Fig 5. This combination produces a noise pattern with complex variations, where fast random fluctuations coexist with slow oscillations, and some harmonic noise components similar to the clean signal are also mixed into the signal. Therefore, the clean signal in Fig 5(b) is significantly degraded, reducing the ability to identify and analyze the signal. After applying the denoising filter, the result shown in Fig 5(d) indicates the effectiveness of the filter, achieving good recovery of the clean signal, preserving the signal shape, and removing the baseline drift. This reflects the ability of the filter to simultaneously handle multiple noise types that vary continuously in practical scenarios. Compared with the single-noise cases, the combined-noise condition is more challenging because the interference contains both broadband and slowly varying components. The visual recovery in Fig 5 therefore supports the ability of the dynamic stage-control mechanism to handle mixed noise conditions without using a fixed number of filtering stages. Although some residual noise remains, the correlation coefficient in the cases shown in Figs 3–5 reaches approximately CC ≈0.97−0.98, indicating that the proposed method preserves the signal morphology and phase very well, which may support subsequent feature extraction or classification stages.

Tables 1–3 show that the proposed method achieves better performance than the other configurations.

**Table 1:** Performance of the proposed filter compared with the conventional LMS filter under Gaussian noise.

${SNR}_{in}$	Filter	MSE	MAE	SNRdB	PSNRdB	CC
+1 dB	LMS Filter	0.001258	0.025752	10.233912	28.356862	0.955078
	Proposed Filter	**0.000730**	**0.021796**	**13.261143**	**31.365796**	**0.976325**
+4 dB	LMS Filter	0.001073	0.021713	10.953085	29.012403	0.961702
	Proposed Filter	**0.000432**	**0.016481**	**15.652598**	**33.644368**	**0.987111**

**Table 2:** Performance of the proposed filter compared with the conventional LMS filter under Pink noise.

${SNR}_{in}$	Filter	MSE	MAE	SNRdB	PSNRdB	CC
+1 dB	LMS Filter	0.001795	0.029940	8.095515	26.651391	0.921638
	Proposed Filter	**0.000755**	**0.021842**	**13.22929**	**31.222869**	**0.976839**
+4 dB	LMS Filter	0.002329	0.030525	7.904127	25.997076	0.917127
	Proposed Filter	**0.000430**	**0.016892**	**16.184195**	**33.660602**	**0.987899**

**Table 3:** Performance of the proposed filter compared with the conventional LMS filter under combined noise.

${SNR}_{in}$	Filter	MSE	MAE	SNRdB	PSNRdB	CC
+1 dB	LMS Filter	0.001051	0.024816	10.958419	29.019102	0.962248
	Proposed Filter	**0.000655**	**0.021117**	**13.785034**	**31.840599**	**0.979549**
+4 dB	LMS Filter	0.001130	0.023013	11.438044	29.122183	0.966004
	Proposed Filter	**0.000411**	**0.016590**	**16.231890**	**33.864578**	**0.988029**

Based on the results in Tables 1–3, compared with the conventional LMS filter, the proposed method reduces the MSE by approximately 37.68% − 81.54%, indicating a substantial reduction in residual reconstruction error after filtering. The MAE decreases by approximately 14.91% − 44.66%, reflecting lower average reconstruction error. In terms of denoising performance, the proposed method achieves an SNR improvement of 2.83 dB - 8.28 dB, while the PSNR increases by 2.82 dB - 7.66 dB compared with the conventional LMS filter. These results indicate that the proposed dynamic-threshold multistage architecture improves denoising performance under the tested simulated noise conditions.

Moreover, when compared with the multistage adaptive denoising filter using a fixed threshold ρ=0.05 [31], the proposed method also shows improved overall performance, as presented in Table 4.

**Table 4:** Performance and execution time of the proposed filter compared with the fixed-threshold multi-stage LMS filter under combined noise.

${SNR}_{in}$	Threshold type	Total filtering stages	Execution time (s)	MSE	MAE	SNRdB	PSNRdB	CC
+4 dB	$\rho_{\text{fixed}}$	4	0.15288	0.000527	0.018594	13.92635	32.28904	0.980217
	$\rho_{\text{automatic}}$	**1**	**0.037812**	**0.000524**	**0.01856**	**14.61347**	**32.80335**	**0.982627**
−1 dB	$\rho_{\text{fixed}}$	2	0.118086	0.001246	0.02932	9.828371	28.21591	0.951935
	$\rho_{\text{automatic}}$	**1**	**0.033873**	**0.001264**	**0.029964**	**10.62957**	**28.98278**	**0.955784**

Note: The reported number of filtering stages includes the final refinement stage. The maximum number of automatically selected Module-I stages is ${\text{J}}_{\text{max}}=\text{3}$. Therefore, a total stage count of four corresponds to three automatically selected Module-I stages plus one final refinement stage.

The results indicate that both MSE and MAE are reduced, while SNR, PSNR, and the correlation coefficient between the clean reference signal and the filtered signal are increased.. Furthermore, based on Table 4, it can be observed that in many cases, the multistage LMS method with a fixed threshold requires more stages to achieve comparable results. In some cases, lowering the fixed threshold can improve the denoising performance. However, the additional improvement is relatively small, while the computational cost increases. These results suggest that the proposed adaptive stopping mechanism based on a dynamic threshold can better balance denoising performance and computational efficiency under the tested noise conditions. Therefore, the proposed multistage LMS adaptive filtering architecture provides a more favorable trade-off between filtering accuracy and execution time than the fixed-threshold approach, making it promising for deployment on resource-constrained devices.

### 4.2. Experimentally acquired data

**Fig 6:**
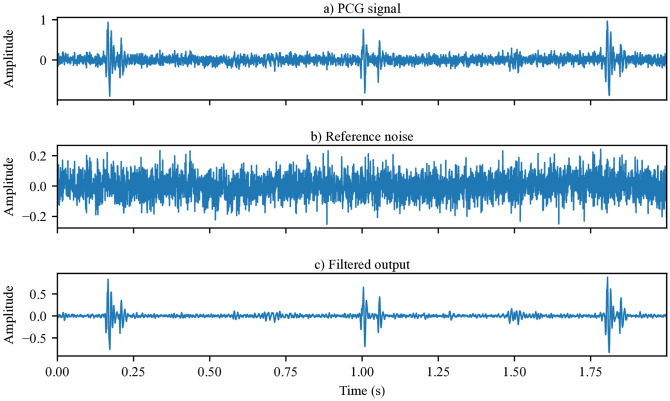
PCG signal.

In addition to using simulated signals as described above to evaluate the filter performance, we also acquired PCG signals in a real-world environment together with the corresponding reference noise signals to serve as inputs to the filter. The results are illustrated in Fig 6, where the original PCG signal in Fig 6(a) contains noticeable background noise, including random noise sources from the environment. In Fig 6(b), after applying the proposed denoising method with a dynamic threshold, the PCG signal shows reduced background noise while retaining the main physiological components of the PCG. The result in Fig 6 shows that the denoising process reduces the background disturbance while preserving the prominent PCG components associated with the main heart sound events. This visual observation is consistent with the reduction in residual-reference correlation and MSC, and with the SQI changes indicating a more concentrated spectrum and preserved physiological signal content.

When a clean reference PCG signal is not available, the effectiveness of the denoising filter is evaluated using two groups of quantitative metrics. The first group consists of noise-reference-based metrics, including the window-based correlation coefficient between the signal channel and the reference channel, as well as magnitude-squared coherence (MSC) in the frequency domain. These metrics provide indirect evidence of the reduction of reference-correlated noise after filtering. The second group consists of Signal Quality Indices (SQI) [47], including excess kurtosis, spectral entropy, and the energy ratio within the physiological frequency band of PCG (20–700 Hz), which are used to assess whether the main physiological characteristics of the PCG signal are retained after filtering.

For the real-world data, the correlated component between the reference channel and the signal channel after filtering is reduced by approximately 99%, decreasing from 0.5197 to 0.004. At the same time, the MSC value is reduced within the physiological PCG frequency band by approximately 75%, suggesting that a large portion of the reference-correlated noise component is attenuated. For the SQI metrics, the results further support the improvement in PCG signal quality after filtering. Specifically, the excess kurtosis increases by approximately 16%, from 22.17 to 26.29, indicating that the amplitude distribution of the filtered signal becomes more peaked, which is consistent with the characteristics of the S1 and S2 components [47]. The normalized spectral entropy decreases from 0.74 to 0.6882, indicating that the spectrum of the filtered signal becomes more concentrated and less affected by broadband noise. In addition, the energy ratio within the physiological PCG frequency band also increases after filtering.

On real-world experimental data, the filter shows promising denoising capability and retains the main physiological features of the PCG signal. However, effective filtering still requires that the acquired reference signal be sufficiently correlated with the noise component to be suppressed.

### 4.3. Algorithmic complexity and computational cost

In the algorithmic architecture of this study, each stage is an M-order filter with the corresponding weight vector. The per-sample computational cost of a typical LMS stage is approximately (2M+1) multiplications or divisions and 2M additions or subtractions per iteration [30]. The automatic stage addition mechanism based on Pearson correlation and the Fisher z transformation relies only on simple statistics and the transformation z=atanh(r), with a standard error of approximately $\text{1}/\sqrt{N_{\text{eff}}-\text{3}}$; therefore, its marginal cost is O(1) per sample, or $O\left(N_{\text{w}}\right)$ per window. These levels of complexity and computational cost indicate that the proposed architecture is suitable for low-latency implementation on devices with limited resources.

In comparison, NLMS requires 3M+1 multiplications and one division [27], the affine projection algorithm (APA) requires $O\left(MK+K^{\text{3}}\right)\;$multiplications or divisions [48], and RLS requires approximately $\text{3}M^{\text{2}}+\text{4}M\;$multiplications or divisions per iteration, corresponding to $O\left(M^{\text{2}}\right)\;$computational complexity [49]. Although these algorithms can provide good denoising performance, they generally require higher computational cost than LMS-based filtering, especially when the filter order or projection order increases. Therefore, the proposed architecture maintains the low per-stage complexity of LMS while reducing unnecessary stage additions through the dynamic stopping mechanism.

### 4.4. Runtime evaluation

The results from Table 4 further indicate the potential of the proposed method with an automatic stopping threshold to reduce latency compared with the fixed-threshold method. The processing time efficiency between the two methods was compared on the same simulated and real-world PCG datasets using the same processing device. Under the tested SNR levels, the dynamic-threshold method achieves an execution time approximately 3.5–4.0 times faster than the fixed-threshold method. This improvement is mainly attributed to: (i) early stopping when the error-reference correlation is no longer statistically significant, and the method is built based on LMS principles (ii) avoiding over-filtering by reducing unnecessary stages.

Furthermore, when tested on real-world acquired signals, the execution time of the proposed denoiser is nearly 2 times faster than that of the fixed-threshold filter. These results suggest that the proposed method can improve computational efficiency and reduce latency under the tested conditions, making it promising for deployment on resource-constrained devices.

### 4.5. Discussion

The results indicate that the proposed dynamic-threshold multistage LMS architecture can improve the trade-off between denoising performance and computational efficiency under the tested conditions. For simulated data, the availability of a clean reference signal allows the use of reference-based metrics such as MSE, MAE, SNR, PSNR, and CC. However, for experimentally acquired PCG data, a clean reference signal is not available; therefore, residual-reference correlation, MSC, and SQI provide indirect evidence of reference-correlated noise reduction and PCG signal preservation.

The effectiveness of the proposed ANC-based method also depends on the availability of a reference signal that is sufficiently correlated with the noise component to be suppressed. In addition, although the proposed method was compared with conventional LMS and fixed-threshold multistage LMS configurations, broader comparisons with NLMS, RLS, APA, wavelet-based methods, and learning-based denoising approaches should be further investigated. Compared with wavelet-based and wavelet-packet denoising methods, such as CEEMD–wavelet denoising [13] and wavelet-packet-based approaches [50,51], the proposed approach does not require the preselection of a mother wavelet, decomposition level, or coefficient-thresholding rule. Wavelet-based methods are effective for non-stationary signal representation and localized noise suppression, but their performance often depends on signal-dependent parameter choices and may require additional tuning when the noise spectrum changes. In contrast, the proposed LMS-based cascade uses a reference-noise channel and a statistical residual-reference stopping criterion to adapt the number of filtering stages online. Therefore, these two families of methods are complementary: wavelet-based approaches are powerful for time-frequency decomposition, whereas the proposed architecture emphasizes low-latency adaptive filtering with automatic stage control.

Future work will also include evaluation under a wider range of input SNR levels, additional signal types such as multitone, chirp, step, and transient signals, repeated random trials for statistical analysis, larger real-world PCG datasets, and validation on embedded hardware platforms.

## 5. Conclusion

In this study, we developed and evaluated a multi-stage adaptive denoising architecture based on the LMS algorithm for signal denoising applications under non-stationary noise conditions. The method is built based on LMS principles and incorporates a correlation-based automatic stage addition mechanism, where the decision threshold is automatically determined via the Fisher-z statistical test and modulated by SNR to determine the necessary number of filter stages in each case. This architecture improves noise reduction capability while reducing unnecessary filtering stages and limiting the risk of over-filtering, which may increase computational cost or affect useful signal characteristics.

The results presented in the Results section indicate the effectiveness of combining a multi-stage adaptive mechanism with statistically based stopping-threshold criteria in signal processing. The method shows improved denoising performance and retains the main signal characteristics, while also indicating potential suitability for resource-constrained devices due to its low computational cost. However, for ANC, the reference noise signal plays a critical role in the effectiveness of the filter. Therefore, future development should further investigate practical methods for acquiring a reference signal that is sufficiently correlated with the noise component in the target signal.

Furthermore, future work will focus on validating the proposed method on embedded devices, extending the framework to other physiological signals such as PPG and ECG, and integrating subsequent feature analysis modules to support downstream biomedical signal assessment.
